# Intercellular adhesion molecule-1 expression and serum levels as markers of pre-clinical atherosclerosis in polycystic ovary syndrome

**DOI:** 10.1186/s13048-019-0566-5

**Published:** 2019-10-19

**Authors:** Nearmeen M. Rashad, Amal S. El-Shal, Hala G. Abomandour, Amr Mostafa Kamel Aboelfath, Mohamed el sayed Rafeek, Mohammad Samir Badr, Ayman E. Ali, Mohammed S. Yousef, Maha Abdelhamid Fathy, Mustafa taha Abdelfattah Sharaf el din

**Affiliations:** 1 Internal Medicine Department, Faculty of Medicine, Zagazig University, Zagazig, Egypt; 2 Medical Biochemistry Department, Faculty of Medicine, Zagazig University, Zagazig, Egypt; 3 Cardiology Department, Faculty of Medicine, Zagazig University, Zagazig, Egypt; 4 Obstetrics and Gynecology Department, Faculty of Medicine, Zagazig University, Zagazig, Egypt; 5 Physiology Department, Faculty of Medicine-Zagazig University, Zagazig, Egypt

**Keywords:** Cardiovascular, Polycystic ovary syndrome, Intercellular adhesion molecule-1, Type 2 diabetes mellitus, Enzyme-linked immunosorbent assay

## Abstract

**Background:**

Polycystic ovary syndrome (PCOS) is a common reproductive endocrine disorder characterized by obesity, hyperandrogenism, and insulin resistance. Intercellular adhesion molecule-1 (ICAM-1) is a proinflammatory and proatherogenic cytokine which is associated with atherosclerosis, insulin resistance, and cardiovascular disease (CVD). The pathogenesis of PCOS is not precisely known. Thus, the purpose of this study was to investigate the potential role of ICAM-1 expression and serum ICAM-1 concentrations in pathogenesis of PCOS. Moreover, we aimed to evaluate the possible relationship between ICAM-1 gene expression with carotid intima-media thickness as well as clinic-morphological features of PCOS.

**Methods:**

This case control study enrolled 180 patients with PCOS and 120 controls groups and they were stratified according to their fasting plasma glucose (FPG) into three subgroups; normal glucose tolerance (NGT) [*n* = 75], those with impaired glucose tolerance (IGT) [*n* = 65], and 40 patients with type 2 diabetes mellitus (T2DM). Circulating ICAM-1 expression levels were determined by real time polymerase chain reaction (RT-PCR). Serum ICAM-1 concentrations were measured using enzyme-linked immunosorbent assay (ELISA).

**Results:**

Our results revealed that PCOS patients had higher values of ICAM-1expression and serum levels. Among PCOS patients, T2DM patients had the highest values of ICAM-1 expression and serum levels compared to IGT and NGT subgroups. The ICAM-1 expression and serum levels were significantly positive correlated with cardiovascular risk and PCOS phenotypes. Linear regression test showed that HOMA-IR was the main predictors of serum ICAM-1 levels in PCOS. Receiver operating characteristic curve (ROC) analysis revealed that, the power of ICAM-1 expression levels was higher than serum ICAM-1 in diagnosis of PCOS and in differentiating T2DM from IGT and NGT subgroups. Interestingly, combination of both ICAM-1 expression and serum levels improved the diagnostic role of serum ICAM-1.

**Conclusion:**

ICAM-1 expression and serum levels were higher in women with PCOS compared to control group also, there was a strong independent association between higher ICAM-1 expression and serum levels with cardiovascular risks in PCOS group.

## Introduction

Polycystic ovary syndrome (PCOS) is the commonest endocrinopathy of premenopausal women characterized by both reproductive and metabolic abnormalities. It affects 5–10% of reproductive-age women [1] Women with PCOS are characterized by chronic anovulation, hyperandrogenism [2], insulin resistance (IR), dyslipidemia, obesity, type 2 diabetes mellitus (T2DM) [3], low-grade chronic inflammation, and increased oxidative stress [4].

The exact pathophysiologic mechanism of PCOS remains still unclear. There is considerable evidence for the involvement of low-grade chronic inflammation and endothelial dysfunction in the pathogenesis of PCOS and its associated features such as insulin resistance (IR), dyslipidemia, and atherosclerosis [5].

Atherosclerosis is an important pathologic cause of cardiovascular diseases (CVD). Additionally, CVD and cerebrovascular diseases are the leading causes of mortality in humans and can have significant impacts on morbidity. Atherosclerosis starts by preclinical increase in the thickness of the internal and medial membrane of the arterial wall which has been related to higher coronary heart disease and stroke rates [6]. Carotid intima–media thickness (CIMT) measured by ultrasound is a noninvasive, safe, low-cost, reproducible, and well validated marker of preclinical atherosclerosis [7, 8].

Intercellular adhesion molecule-1 (ICAM-1), (CD54), a 90- kDa cell surface glycoprotein, is involved in the firm adhesion of leukocytes to endothelium and their trans-endothelial migration to sites of inflammation [9]. An important event in the initiation of atherosclerosis is the adhesion of circulating monocytes to the activated endothelial cells and their subsequent transendothelial migration into the subendothelial space. These processes depend on the coordinated expression and activation of several cellular adhesion molecules (CAMs) such as vascular cell adhesion molecule1 (VCAM-1), ICAM-1, E-selectin and their counter-receptors on monocytes [10]. Meanwhile, the expression of endothelial cell adhesion proteins is induced by proinflammatory cytokines such as tumor necrosis factor- α (TNF-α) and interleukin-1 (IL-1β) and by specific oxidants [10, 11].

The concept of the critical role of adhesion molecules in endothelial dysfunction has been reported [12]. It has been shown recently that cellular adhesion molecule levels, including ICAM-1 suggested to be increased in patients with PCOS [13].

PCOS is a highly complex endocrine disorder and phenotypically is heterogeneous. The purpose of this study was to investigate the potential role of ICAM-1 serum and expression levels. Moreover, we aimed to explore role of ICAM-1 in early detection of cardiovascular risk in patients with PCOS, and to evaluate the possible relationship between ICAM-1 gene expression with carotid intima-media thickness as well as clinic-morphological features of PCOS.

## Subject and method

This case control study included 300 unrelated subjects. One hundred eighty PCOS patients recruited from Outpatient Clinics of the Endocrinology Unit of Internal Medicine and Obstetrics and Gynecology Departments, Faculty of Medicine, Zagazig University, Egypt and 120 healthy women matched to PCOS cases as regard age, and body mass index (BMI). PCOS patients were then stratified into three subgroups according to their fasting plasma glucose (FPG) based on the American Diabetes Association criteria reported in 2016, [14] those with normal glucose tolerance (NGT; 75 women), those with impaired glucose tolerance (IGT; 65 patients), and 40 patients with type 2 diabetes mellitus (T2DM). The diagnosis of PCOS was based on the 2004 revised Rotterdam criteria [15]. All patients were subjected to thorough history taking, full clinical assessment, and anthropometric measures of obesity. Ovarian volume and antral follicular count (AFC) were evaluated by transvaginal ultrasound (TVS). Body compositions including fat mass (FM) and fat free mass (FFM) were measured by Dual-energy X-ray absorptiometry (DEXA).

Exclusion criteria for all women included a history of hyperandrogenic states (such as non-classical congenital adrenal hyperplasia, androgen secreting tumors, Cushing’s syndrome, 21-hydroxylase deficiency, or hyperprolactinemia), diabetes mellitus (DM), hypertension, liver, kidney, or thyroid diseases.

### Ethics approval and consent to participate

A written informed consent was taken from all of the participants after explaining details and benefits as well as risks to them. The ethical committee of Faculty of Medicine, Zagazig University approved this study.

### Sampling of blood

The blood samples of all study’s subjects were drawn after an overnight fast and divided into 3 portions: 1 ml of whole blood was collected into EDTA tubes, for RNA extraction and HbA1c; 1 ml of blood was collected into potassium oxalate and sodium fluoride containing tubes for fasting blood glucose (FBG) and 2-h plasma glucose. Sera were separated from remaining sample part and stored at − 20 °C until analysis.

### Biochemical analysis

We determined FBG and 2-h plasma glucose levels using the glucose oxidase method (Spinreact, Girona, Spain). Total cholesterol, HDL cholesterol, and triglycerides levels were measured by routine enzymatic methods (Spinreact, Girona, Spain). The LDL cholesterol level was calculated using the Friedewald formula [16].

### Immunochemical assays

Fasting serum insulin (FSI) levels were determined by high-sensitivity linked immunosorbent assay (ELISA) kit provided by (Biosource Europe S.A., Nivelles, Belgium). Homeostasis model assessments of insulin resistance (HOMA-IR) and В-cell function (HOMA-B) were calculated. Serum ICAM-1 concentrations were measured with a sandwich ELISA using the human RayBio® Human sICAM-1 ELISA kit (RayBio, Nacross, USA).

### RNA extraction, cDNA synthesis and real time PCR for ICAM-1 mRNA gene expression

Total RNA was extracted from whole blood using QIAamp® RNA Blood MiniKit following the manufacturer’s protocol (QIAGEN, Valencia,USA). The quantity and purity of RNA was confirmed by optical density (OD) at 260 and 280 nm using an ultraviolet spectrometer, with acceptable RNA purity ranging from 1.8 to 2.1. The ratio of absorbance values at 260 and 280 nm indicated an estimate of RNA purity. The extracted RNA was reverse transcribed by (QuantiTect Reverse Transcription Kit, QIAGEN, Valencia, CA, USA) as recommended by the manufacturer. Expression levels of *ICAM-1* mRNA were determined by quantitative real time PCR (RT-PCR) according to previous study [17]. The ICAM-1 primers sequence were: forward primer; 5′- AGGCCACCCCAGAGGACA AC − 3′ and reverse primer 3′- CCCATTATGACTGCGGCTGCTA − 5′, the product was 406 bp; a housekeeping gene β-actin forward primer: 5′-ATGTTTGAGACCTTCAACAC − 3′, and reverse primer: 5′-CACGTCACACTTCATGATGG-3′, of 489 bp length; was used as an internal control. The expression of candidate gene mRNA was measured using a StepOne™ System (Applied Biosystems). The PCR was performed in 25 final reaction volume using QuantiTect SYBR® Green PCR Kits (QIAGEN, Valencia, CA, USA) containing 12.5 μl 2X SYBR Green PCR Master Mix, 1 μM of each primer and 2 μl cDNA. The following conditions were used: denaturation at 95 °C for 5 min (hot start), 40 cycles of denaturation (95 °C for 10 s), and combined annealing and extension annealing (60 °C for 30 s). The expression of ICAM-1 was reported as the ΔCt value, which was calculated by subtracting the CT values of β-actin from the CT values of the target ICAM-1.

### Statistical analysis

Data analyses were done with statistical package for the social sciences software (SPSS Version 21, Chicago, Illinois). Data were expressed as mean ± standard deviation (SD). The relationships of ICAM-1 serum and expression level with clinical and laboratory parameters among PCOS patients were tested with the Pearson correlation. Receiver operating characteristic (ROC) analysis was performed to assess the diagnostic power of ICAM-1 serum and expression level. Linear regression analysis was done to detect the main predictors of serum ICAM-1 in PCOS group. We considered *P* to be significant at < 0.05 with a 95% confidence interval (CI).

## Results

### Clinical and laboratory characteristics of the study subjects

In PCOS group, patients had significantly higher values of systolic blood pressure (SBP), total cholesterol (TC), triglycerides (TG), low-density lipoprotein (LDL)-cholesterol, FPG, 2-h plasma glucose, FSI, hemoglobin A1c (HbA1c), homeostatic model assessment-IR (HOMA-IR), high sensitivity C-reactive protein (hsCRP), white blood cell (WBC) count, and CIMT when compared to control group. Additionally, as expected patients with PCOS had significantly higher values of clinical and biochemical characteristic of PCOS; hirsutism score, ovarian volume, AFC, FSH, LH, LH/FSH, DHEA-S, androstenedione, total testosterone,and FAI compared control group. On the contrary, patients with PCOS had significantly lower levels of HDL, HOMA –β, and SHBG when compared with controls (Table 1).

**Table 1:** Clinical, anthropometric and laboratory characteristics of studied groups

	Control group (mean ± SD) (*n* = 120)	PCO patients (mean ± SD) (*n* = 180)	P
Age (years)	31.38 ± 7.88	31.95 ± 7.42	0.527
Systolic blood pressure (mm Hg)	127.6 ± 5.62	131.06 ± 7.021	<0.001^*^
Diastolic blood pressure (mm Hg)	86.2 ± 3.94	87.31 ± 6.074	0.076
Hirsutism score	5.53 ± 0.472	13.54 ± 2.51	<0.001^*^
Body mass index (kg/m2)	30.28 ± 57	31.889.99	0.103
Waist/hip ratio	1.11 ± 0.26	1.14 ± 0.30	0.392
FMI (kg/m2)	7.66 ± 1.45	7.86 ± 1.579	0.260
FFMI (kg/m2)	21.81 ± 4.13	22.42 ± 4.47	0.233
Ovarian volume	4.33 ± 1.1	10.64 ± 1.3	<0.001^*^
AFC	5.33 ± 1.1	12.14 ± 1.4	<0.001^*^
Total cholesterol (mg/dL)	179.47 ± 19.7	198.8 ± 52.38	<0.001^*^
Triglycerides (mg/dL)	150.23 ± 20.84	187.6 ± 62.14	<0.001^*^
LDL cholesterol (mg/dL)	117.48 ± 19.05	165.98 ± 60.15	<0.001^*^
HDL cholesterol (mg/dL)	56.58 ± 6.04	39.2 ± 13.63	<0.001^*^
Fasting plasma glucose (mg/dL)	87.65 ± 9.14	108.2 ± 21.62	<0.001^*^
2-h plasma glucose (mg/dL)	127.6 ± 24.2	163.67 ± 41.7	<0.001^*^
HbA1c (%)	5.82 ± 0.182	6.35 ± 0.977	<0.001^*^
Fasting serum insulin (lU/mL)	7.14 ± 1.427	20.05 ± 7.3	<0.001^*^
HOMA-IR	1.55 ± 0.39	4.63 ± 1.87	<0.001^*^
HOMA-β	169.7 ± 18.14	122.01 ± 43.6	<0.001^*^
FSH (mIU/mL)	6.17 ± 1.53	6.6 ± 1.26	<0.001^*^
LH (mIU/mL)	6.95 ± 0.646	10.3 ± 1.23	<0.001^*^
LH/FSH	1.24 ± 0.54	1.6 ± 0.278	<0.001^*^
SHBG (nmol/L)	53.58 ± 6.04	28.8 ± 4.71	<0.001^*^
DHEA-S (mg/mL)	1.22 ± 0.33	2.37 ± 0.316	<0.001^*^
Androstenedione (ng/mL)	1.41 ± 0.508	1.62 ± 0.557	<0.001^*^
Total testosterone (ng/mL)	0.67 ± 0.18	1.1 ± 0.218	<0.001^*^
Free androgen index	1.03 ± 0.36	2.56 ± 1.22	<0.001^*^
hs-CRP (μg/ml)	2.61 ± 0.51	5.56 ± 0.98	<0.001^*^
WBC count (cell×10^3^/μl)	4.74 ± 0.397	6.97 ± 2.31	<0.001^*^
CIMT (mm)	0.69 ± 0.06	1.18 ± 0.46	<0.001^*^
Serum ICAM-1 (ng/ml)	87.8 ± 49.5	445.17 ± 184.95	<0.001^*^
Relative ICAM expression levels	0.75 ± 0.48	4.57 ± 2.16	<0.001^*^

*FSI* fasting serum insulin, *FPG* fasting plasma glucose, *AFC* antral follicle cells, *FMI* fat mass index, *FFMI* fat free mass index, *HOMA-IR* homeostasis model assessments of insulin resistance, *DHEA* dehydroepiandrosterone;. *P < 0.05 when compared with control group

### Clinical and laboratory characteristics of women with PCOS

Among PCOS group, T2DM patients had significant higher values of body composition parameters; waist/hip ratio, FMI% and FFMI%. Also, systolic and diastolic blood pressure as well as, TG, FPG, 2-h plasma glucose, FSI, HbA1c, HOMA-IR, hs-CRP, WBC count, and CIMT values were increased when compared to NGT and IGT groups. In addition, PCOS phenotypes; ovarian volume, AFC, total testosterone, FAI, LH and DHEA-S were significantly higher in T2DM patients compared to NGT and IGT PCO subgroups. On the contrary, we detected significant lower SHBG and HOMA-β levels in T2DM patients than in those NGT or IGT patients.

In IGT subgroup, we observed significant higher levels of systolic and diastolic blood pressure as well as waist/hip ratio, FMI%, FFMI%, FPG, 2-h plasma glucose, FSI, HbA1c, HOMA-IR, hs-CRP, and CIMT s than in those NGT group. Moreover, PCOS phenotypes values; ovarian volume, AFC, total testosterone, FAI, FSH, LH, LH/FSH and DHEA-S were significantly higher in IGT than NGT. On the other hand, we demonstrated significant lower values of SHBG, HDL, and HOMA-B in IGT compared to NGT (Table 2).

**Table 2:** Clinical, anthropometric and laboratory characteristics of PCOS groups

Parameters	PCO patients (n = 180)		
	NGT Group (mean ± SD) (*n* = 75)	IGT Group (mean ± SD) (*n* = 65)	T2DM Group (mean ± SD) (*n* = 40)
Systolic blood pressure (mm Hg)	127.76 ± 7.21	134.49 ± 4.74^a^	139.67 ± 7.04^b,c^
Diastolic blood pressure (mm Hg)	83.8 ± 3.86	87.85 ± 6.33^a^	90.98 ± 5.80^b,c^
Hirsutism score	13.78 ± 2.594	13.42 ± 2.9	13.29 ± 1.42
Body mass index (kg/m2)	32.96 ± 14.25	30.11 ± 4.41	32.65 ± 5.73
Waist/hip ratio	0.96 ± 0.212	1.34 ± 0.27^a^	1.17 ± 0.27^b,c^
Ovarian volume	9.88 ± 0.491	10.75 ± 1.21^a^	11.89 ± 1.4^b,c^
AFC	11.38 ± 0.491	12.25 ± 1.17^a^	13.39 ± 1.6^b,c^
FMI (kg/m2)	6.20 ± 0.68	8.7 ± 0.63^a^	9.49 ± 0.63^b,c^
FFMI (kg/m2)	17.65 ± 1.96	25.02 ± 1.82^a^	27.17 ± 1.06^b,c^
Total cholesterol (mg/dL)	191.21 ± 54.43	201.5 ± 54.43	209.02 ± 43.32
Triglycerides (mg/dL)	174.02 ± 55.3	184.2 ± 57.19	216.04 ± 73.1^b,c^
LDL cholesterol (mg/dL)	168.15 ± 61.11	163.17 ± 57.96	166.46 ± 63.11
HDL cholesterol (mg/dL)	41.46 ± 11.78	36.21 ± 13.85^a^	39.8 ± 15.8
FPG (mg/dL)	91.1 ± 6.84	108.16 ± 12.50^a^	140.5 ± 12.7^b,c^
2-h blood glucose (mg/dL)	130.28 ± 23.2	171.94 ± 28.68^a^	212.8 ± 30.1^b,c^
FSI (lU/mL)	13.5 ± 3.289	26.96 ± 5.51^a^	21.1 ± 3.34^b,c^
HOMA-IR	2.85 ± .855	6.25 ± 1.44^a^	5.34 ± 0.647^b,c^
HOMA-β	139.23 ± 54.11	124.4 ± 35.3^a^	108.6 ± 41.55^b,c^
HbA1c (%)	5.66 ± 0.35	6.37 ± 0.615^a^	7.62 ± 0.983^b,c^
Total testosterone (ng/mL)	0.9 ± 0.231	0.99 ± 0.304^a^	1.07 ± 0.155^b,c^
Free androgen index	1.813 ± 0.98	2.88 ± 0.81^a^	3.4 ± 0.69^b,c^
FSH (mIU/mL)	6.02 ± 1.314	7.107 ± 1.06^a^	7.05 ± 0.985^b^
LH (mIU/mL)	9.45 ± 0.491	10.44 ± 1.15^a^	11.98 ± 0.201^b,c^
LH/FSH	1.63 ± 0.341	1.48 ± 0.144^a^	1.73 ± 0.241^c^
SHBG (nmol/L)	33.54 ± 3.108	26.23 ± 2.011^a^	24.197 ± 0.96^b,c^
DHEA-S (mg/mL)	2.17 ± 0.245	2.43 ± 0.241^a^	2.66 ± 0.286^b,c^
Androstenedione (ng/mL)	1.23 ± 0.371	1.91 ± 0.539^a^	1.88 ± 0.43
hs-CRP (μg/ml)	4.92 ± 0.761	5.79 ± 0.760^a^	6.37 ± 0.91^b,c^
WBC count (cell×10^3^/μl)	4.79 ± 0.713	8.45 ± 2.27^a^	8.58 ± 1.281^b,c^
CIMT (mm)	0.96 ± 0.263	1.09 ± 0.36^a^	1.74 ± 0.467^b,c^
Serum ICAM-1 (ng/ml)	281.3 ± 95.29	509.4 ± 82.16^a^	594.6 ± 156.12^b,c^
Relative ICAM expression levels	2.68 ± 0.85	5.7 ± 1.52^a^	6.05 ± 2.119^b,c^

*NGT* Normal glucose tolerance, *IGT* impaired glucose tolerance, *T2DM* type 2 diabetes mellitus, *FSI* fasting serum insulin, *FPG* fasting plasma glucose, *AFC* antral follicle cells, *FMI* fat mass index, *FFMI* fat free mass index, *HOMA-IR* homeostasis model assessments of insulin resistance, *DHEA* dehydroepiandrosterone

^a^ significant difference between IGT vs NGT

^b^ significant difference between T2DM vs NGT.,^c^ significant difference between T2DM vs IGT,

### Comparison of expression and serum levels of ICAM-1 in studied groups

**Fig. 1:**
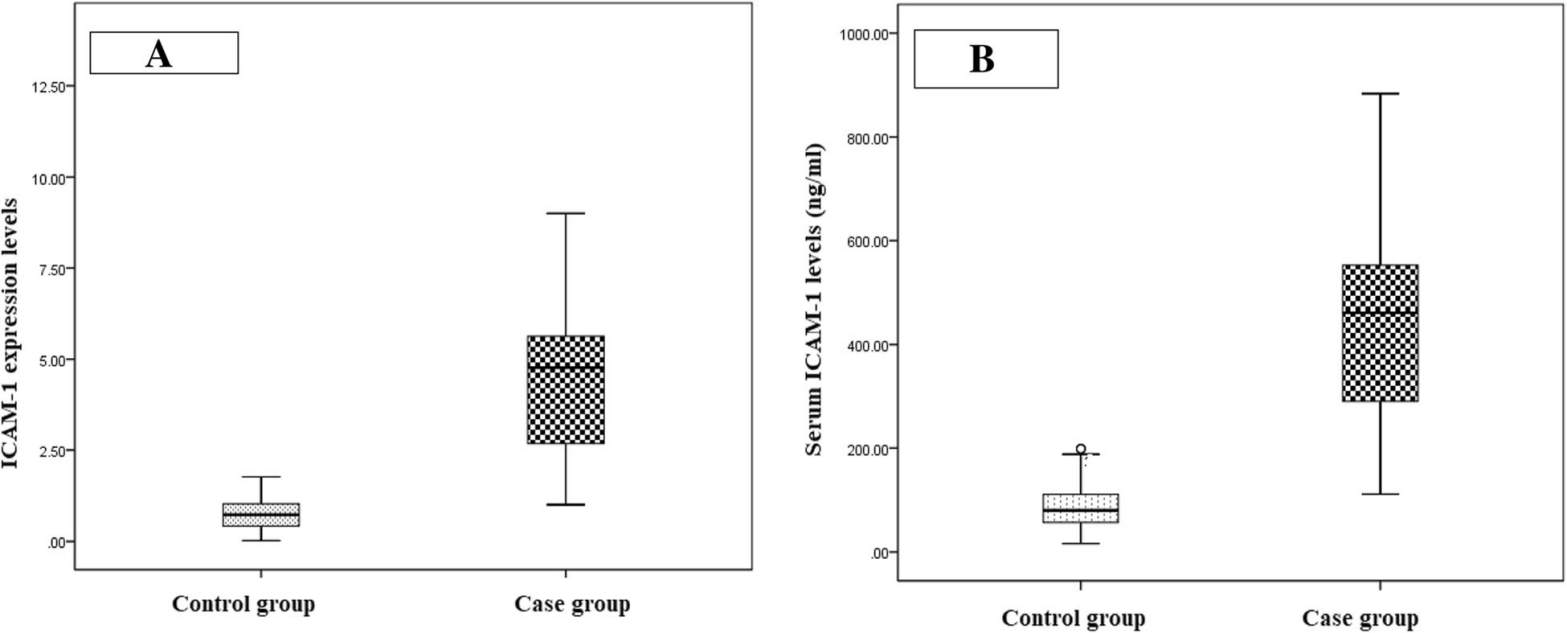
**a** Comparison of ICAM-1 expression levels (ng/ml) levels in studied groups. According to the study, the difference of ICAM-1 expression levels were statistically significant higher in PCOS patients compared to healthy controls. **b**.Comparison of serum ICAM-1 levels in studied groups. Current study revealed that the ICAM-1 levels were statistically significant higher in PCOS patients compared to healthy controls

**Fig. 2:**
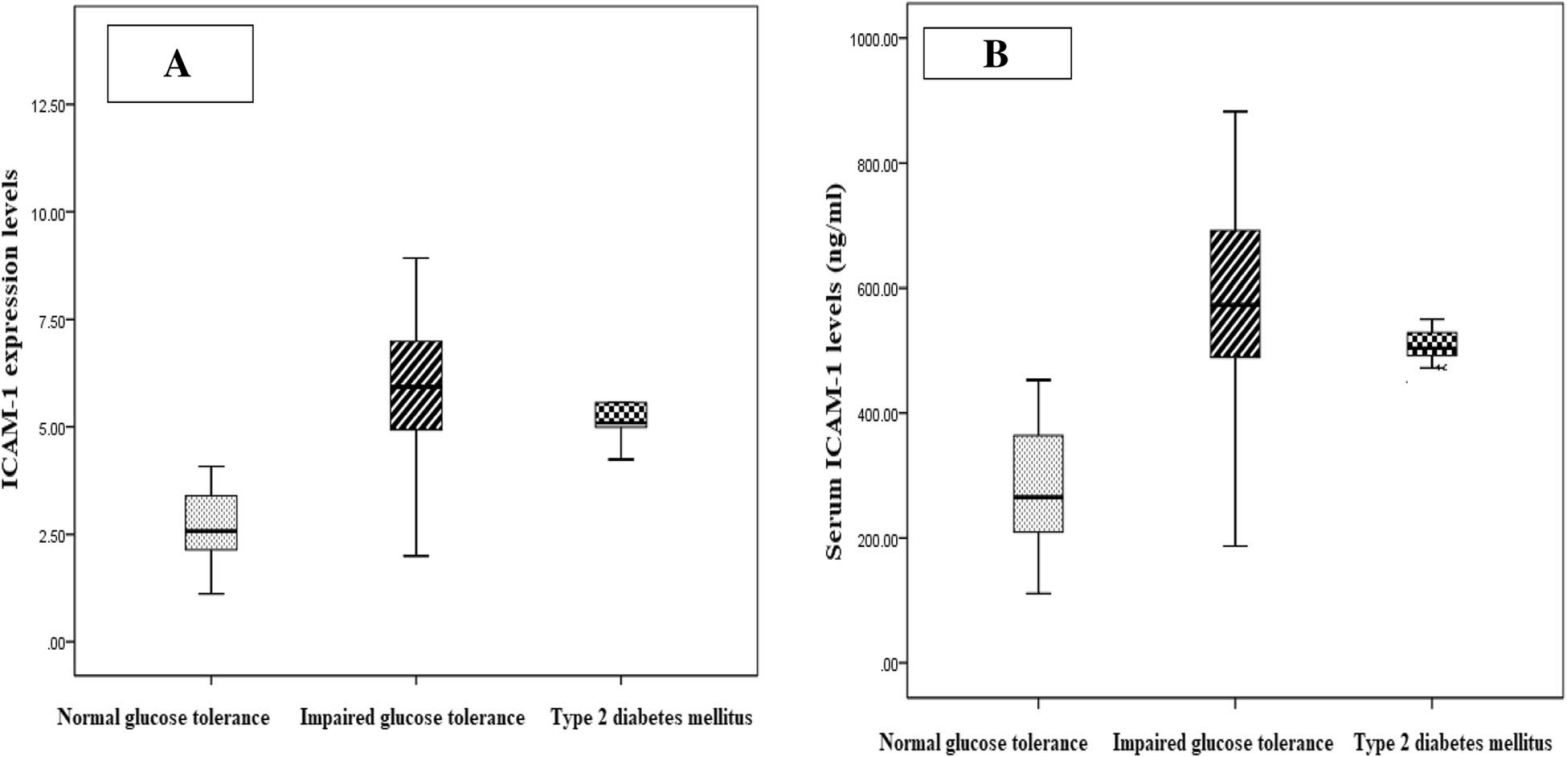
**a** Comparison of ICAM-1 expression levels in PCOS groups. Our study found that the ICAM-1 expression levels were statistically significant higher in PCOS patients with IGT compared to other PCOS groups. **b** Comparison of serum ICAM-1 levels (ng/ml) levels in PCOS groups. Our study found that the ICAM-1 serum levels were statistically significant higher in PCOS patients with IGT compared to other PCOS groups

Regarding expression levels of ICAM-1, PCOS patients (4.57 ± 2.16) had significantly higher levels compared to control group (0.75 ± 0.48) (Fig. 1a). Among PCOS group, T2DM patients (6.05 ± 2.119) had significantly higher expression levels of ICAM-1 compared to IGT (5.7 ± 1.52) and NGT (2.68 ± 0.85) (Fig. 2a).

Regarding serum ICAM-1 levels, PCOS patients (445.17 ± 184.95 ng/ml) had significantly higher levels compared to control group (87.8 ± 49.5 ng/ml) (Fig. 2a). Among PCOS group, T2DM patients (594.6 ± 156.12) had significantly higher serum levels of ICAM-1 compared to IGT (509.4 ± 82.16 ng/ml) and NGT (281.3 ± 95.29 ng/ml) (Fig. 2b). In addition, IGT group had significantly higher expression and serum levels of ICAM-1 compared to NGT group (Fig. 1B and 2B).

### Correlation between serum and expression levels of ICAM-1 with clinical and biochemical parameters of PCOS patients

In PCOS group, (*n* = 180), serum ICAM-1 *levels* were significantly positive correlated with of body composition parameters; BMI, waist/hip ratio, FMI% and FFMI. Furthermore, serum ICAM-1 levels were significantly positive correlated with PCOS markers; ovarian volume, AFC, total testosterone, FAI, FSH, LH, LH/FSH, DHEA-S, and androstenedione. Even more interestingly, serum ICAM-1 levels were significantly positive correlated with cardio metabolic factors; FPG, 2-h blood glucose, FSI, HOMA-IR, HbA1c, hs-CRP, WBC count and CIMT. On the contrary, serum ICAM-1 levels were negatively correlated with HOMA-β and SHBG (Table 3).

**Table 3:** Pearson correlation of serum ICAM-1 (ng/ml) and ICAM-1 expression levels with clinical, anthropometric as well as biochemical characteristics in PCOS group

Characteristics	Serum ICAM-1 levels		ICAM-1 expression levels	
	r	p	r	p
Hirsutism score	0.095	0.205	0.095-	0.210
Body mass index (kg/m2)	0.086	0.253	0.073	0.332
Waist/hip ratio	0.494	<0.001^*^	0.501	<0.001^*^
Ovarian volume	0.187	0.012*	0.180	0.016
AFC	0.187	<0.001^*^	0.180	<0.001^*^
FMI%	0.689	<0.001^*^	0.709	<0.001^*^
FFMI%	0.677	<0.001^*^	0 .666	<0.001^*^
Total cholesterol (mg/dL)	0.111	0.137	0.114	0.128
Triglycerides (mg/dL)	0.019	0.797	0.030	0.694
LDL cholesterol (mg/dL)	0.014	0.853	0.008	0.912
HDL cholesterol (mg/dL)	0.036	0.631	0.044	0.561
FPG (mg/dL)	0.435	<0.001^*^	0.477	<0.001^*^
2-h blood glucose (mg/dL)	0.471	<0.001^*^	0.438	<0.001^*^
FSI (lU/mL)	0.854	<0.001^*^	0.794	<0.001^*^
HOMA-IR	0.874	<0.001^*^	0.932	<0.001^*^
HbA1c (%)	0.404	<0.001^*^	0.369	<0.001^*^
HOMA-β	−0.147	<0.001^*^	0.222	<0.001^*^
Total testosterone (ng/mL)	0.114	<0.001^*^	0.121	<0.001^*^
Free androgen index	0.466	<0.001^*^	0.469	<0.001^*^
FSH (mIU/mL)	0.502	<0.001^*^	0.518	<0.001^*^
LH (mIU/mL)	0.549	<0.001^*^	0.561	<0.001^*^
SHBG (nmol/L)	−0.658	<0.001^*^	0.639	<0.001^*^
DHEA-S (mg/mL)	0.269	<0.001^*^	0.262	<0.001^*^
Androstenedione (ng/mL)	0.613	<0.001^*^	0.625	<0.001^*^
hs-CRP (μg/ml)	0.342	<0.001^*^	0.374	<0.001^*^
WBC count (cell×103/μl)	0.602	<0.001^*^	0.650	<0.001^*^
CIMT (mm)	0.252	<0.001^*^	0.257	<0.001^*^

*FSI* fasting serum insulin, *FPG* fasting plasma glucose, *AFC* antral follicle cells, *FMI* fat mass index, *FFMI* fat free mass index, *HOMA-IR* homeostasis model assessments of insulin resistance, *DHEA-S* dehydroepiandrosteron sulfate e;. *P < 0.05 when compared with control group

Regarding ICAM-1expression levels, there were significantly positive correlation with BMI, waist/hip ratio, FMI% and FFMI, AFC, FPG, 2-h blood glucose, FSI, HOMA-IR, HbA1c, total testosterone, FAI, FSH, LH, LH/FSH, DHEA-S, and androstenedione, hs-CRP, WBC count and CIMT. On the contrary, the expression levels of ICAM-1 were negatively correlated with HOMA-β and SHBG (Table 3*).*

### Linear regression analysis with serum ICAM-1 levels as dependent variable in PCOS groups

In PCOS group, linear regression analysis revealed that only HOMA-IR was the main predictor of serum ICAM-1 levels among other clinical and laboratory biomarkers of PCOS (Table 4).

**Table 4:** linear regression analyses in PCOS women to test the influence of the main independent variables against serum ICAM-1 levels (dependent variable) in PCOS women

Model		Unstandardized Coefficients		Standardized Coefficients	t	*P* value	95% C.I.	
		B	SE	Beta			Lower Bound	Upper Bound
1	(Constant)	135.851	80.832		1.681	0.095	−23.727-	295.428
	Total cholesterol	−0.019-	0.103	0.006-	0.190	0.850	−0.222-	0.183
	LDL cholesterol	−0.001-	0.087	0.000	0.014	0.989	−0.172-	0.170
	FPG	−0.163-	0.456	0.019	0.357	0.721	−1.063-	0.737
	HOMA-IR	90.239	4.100	0.917	22.008	< 0.001*	82.144	98.334
	Body mass index	−0.215-	0.537	0.012-	0.400	0.689	−1.274-	0.844
	LH	1.267	7.087	0.008	0.179	0.858	−12.725-	15.259
	hs-CRP	−4.453-	7.328	0.024	0.608	0.544	−18.919-	10.014
	WBC count	2.683	3.020	0.034	0.888	0.376	−3.280-	8.645
	Ovarian volume	−7.053-	5.234	0.050	1.347	0.180	−17.386-	3.280
	Hirsutism score	−2.981-	2.232	0.040	1.336	0.184	−7.388-	1.426
	CIMT	23.455	15.458	0.059	1.517	0.131	−7.061-	53.972

### Linear regression analysis with ICAM-1 expression levels as dependent variable in PCOS groups

In PCOS group, linear regression analysis showed that HOMA-IR, WBC count, and ovarian volume were the main predictors of ICAM-1 expression levels among other clinical and laboratory biomarkers of PCOS (Table 5).

**Table 5:** linear regression analyses in PCOS women to test the influence of the main independent variables against ICAM-1 gene expression (dependent variable) in PCOS women

Model		Unstandardized Coefficients		Standardized Coefficients	t	*P* value	95% C.I.	
		B	SE	Beta			Lower Bound	Upper Bound
1	(Constant)	−0.928	1.221		0.760	0.448	−3.340	1.483
	Total cholesterol	−0.001	0.002	0.017	0.456	0.649	−0.004	0.002
	LDL cholesterol	0.000	0.001	0.012	0.340	0.734	−0.002	0.003
	FPG	0.004	0.007	0.036	0.526	0.599	−0.010	0.017
	HOMA-IR	0.829	0.062	0.720	13.389	< 0.001*	0.707	0.952
	Body mass index	0.000	0.008	0.002	0.052	0.958	−0.016	0.016
	LH	0.114	0.107	0.065	1.069	0.287	−0.097	0.326
	hs-CRP	0.139	0.111	0.063	1.251	0.213	−0.080	0.357
	WBC count	0.152	0.046	0.162	3.328	0.001*	0.062	0.242
	Ovarian volume	−0.178-	0.079	0.0107	2.247	0.026*	−0.334	−0.022
	Hirsutism score	0.012	0.034	0.014	0.368	0.713	−0.054	0.079
	CIMT	0.039	0.234	0.008	0.168	0.867	−0.422	0.500

### Accuracy of serum and expression levels of ICAM-1 for diagnosis of PCOS by ROC analysis

**Fig. 3:**
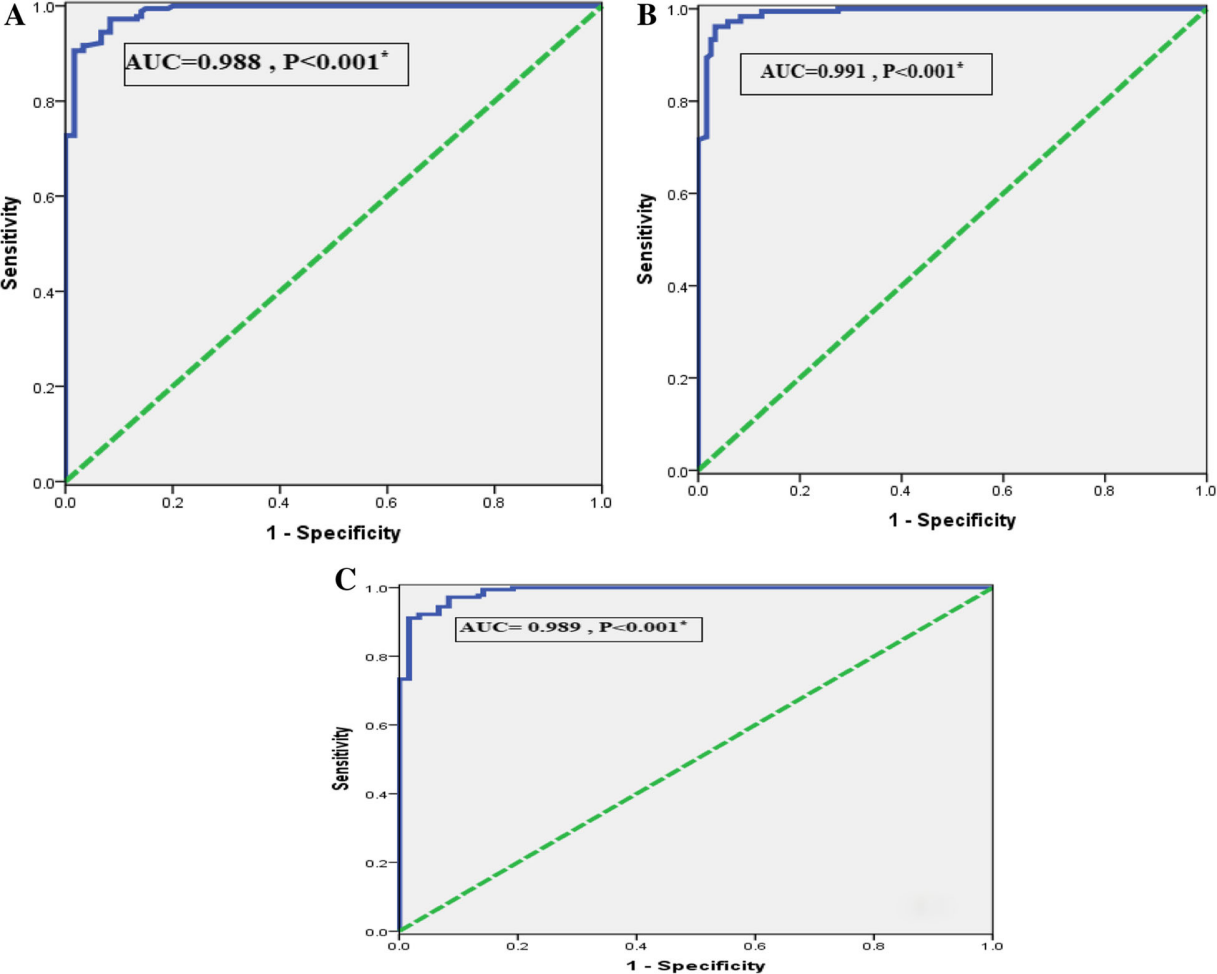
**a** ROC curve of serum ICAM-1 for diagnosis PCOS. **b** ROC curve of ICAM-1 expression levels for diagnosis PCOS patients. **c** ROC curve for combination of ICAM-1 expression and serum levels for diagnosis PCOS patients

The power of serum ICAM-1 (ng/ml) levels to diagnose PCOS among studied subjects was evaluated using ROC analysis. The AUC was 0.988 (95% CI = 0.980–0.997) with sensitivity = 99.4%, specificity = 85%, and the cutoff values (121.2). When compared PCO patients to control, (Fig. 3a).

Regarding relative expression levels of ICAM-1, the AUC to diagnose PCOS among studied subjects was 0.991 (95% CI = 0.983–0.999) with sensitivity = 99.4%, specificity = 87.5%, and the cutoff values (1.11), (Fig. 3b).

ROC analysis revealed that combined serum ICAM-1 and expression levels to discriminate PCOS from control, the AUC was 0.989 (95% CI 5 0.980–0.997, *P* < 0.001) with sensitivity = 99.4%, specificity = 85.8%, (Fig. 3c).

### Accuracy of serum and expression levels of ICAM-1 for discriminating T2DM from NGT PCOS patients by ROC analysis

**Fig. 4:**
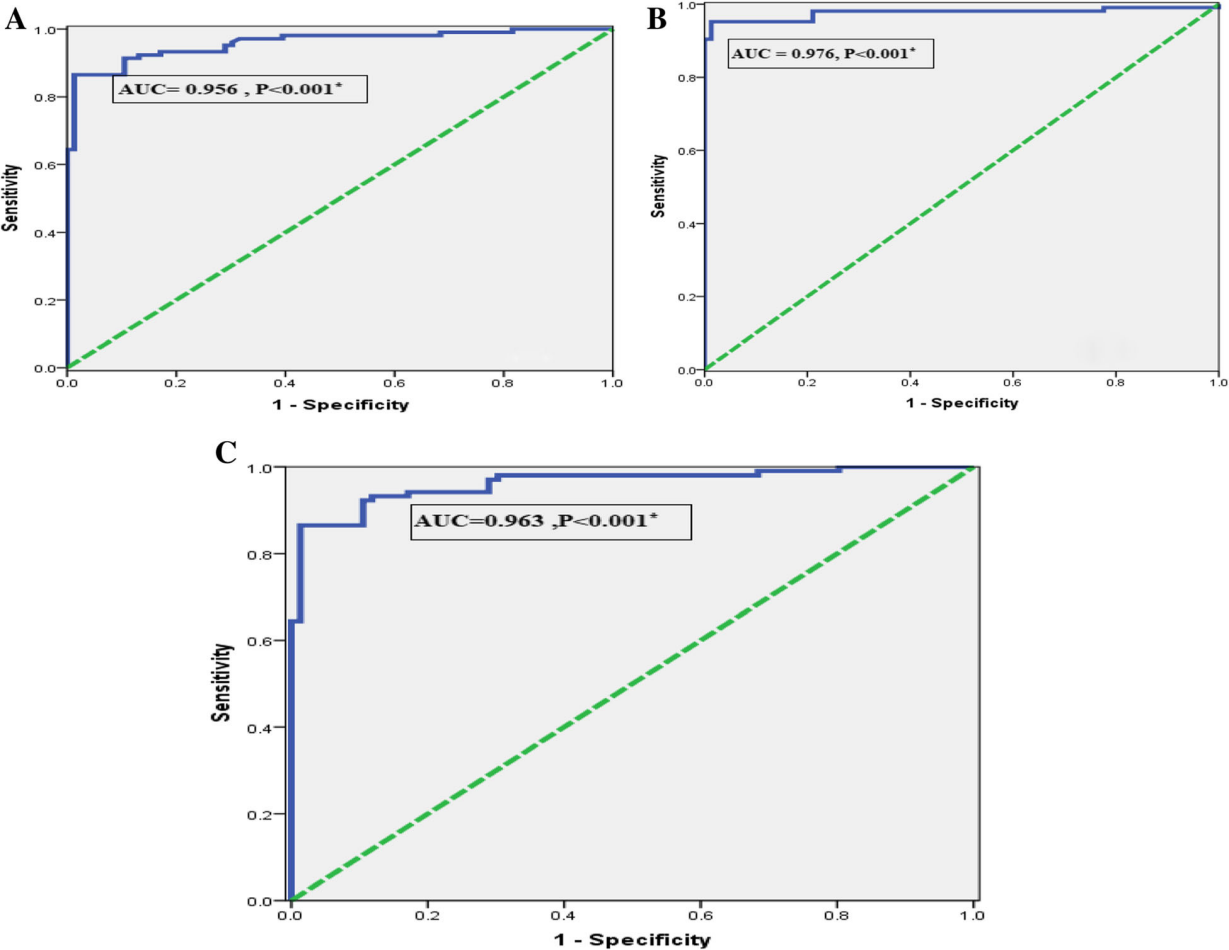
**a** ROC curve of serum ICAM-1 for discriminating T2DM from NGT patients with PCOS*.* **b** ROC curve of serum ICAM-1 for discriminating T2DM from NGT patients with PCOS. **c** ROC curve for combination of ICAM-1 expression and serum levels for discriminating T2DM from NGT patients with PCOS

Among PCOS patients, the power of serum ICAM-1 (ng/ml) levels in discriminating T2DM from NGT, the AUC was 0.956 (95% CI = 0.933–0.986) with sensitivity = 93.3%, specificity = 82.9%, and the cutoff values (121.2). (Fig. 4a).

Regarding relative expression levels of ICAM-1, the AUC to diagnose PCOS among studied subjects was 0.976 (95% CI = 0.951–1.000) with sensitivity = 95.2%, specificity = 99.3%, and the cutoff values (1.11), (Fig. 4b).

ROC analysis revealed that combined serum and expression levels of ICAM-1 to discriminate PCOS from control, the AUC was 0.963 (95% CI = 0.937–0.988, *P* < 0.001) with sensitivity = 98.1%, specificity = 69.9% (Fig. 3c).

## Discussion

Because current treatment of PCOS is still suboptimal, and cardio-metabolic diseases is the common cause of death all over the world among PCO cases, early assessment of levels of cytokines could potentially help in early diagnosis and prevention of these common diseases To the best of our knowledge, this is the first study regarding for the association between ICAM-1serum level and gene expression as cardiovascular predictors with PCOS as well as its clinic-morphological features.

In order to assess the risk of cardio-vascular disease among PCOS, our results revealed that the cardio-metabolic factors; systolic blood pressure, TC, TG, LDL cholesterol, 2-h plasma glucose, FSI, HbA1c, HOMA-IR,,FPG and CIMT were significantly higher in PCOS compared to control group. Our previous experience also suggested that PCOS women had higher values of parameters of hyperglycemia and dyslipidemia as well as fat mass [18, 19].

Similar to our results, Glueck et al. found that PCOS patients are at an increased risk of metabolic syndrome and cardiovascular risk factors in later life [20]. Similar results confirmed by, Zhao et al. they observed high risk of coronary heart disease in PCOS [21]. Moreover, Morgan et al. study detected higher mortality in PCOS compared to control group [22].. On the contrary, Pierpoint et al. [23] and Wild et al. [24] conducted their studies on CAD morbidity or mortality among PCOS patients, and they did not find any significant association between PCOS and CAD morbidity and/or mortality.

Regarding lipid profile in PCOS women, similar to our resultsWild et al. Showed that women with PCOS have higher levels of TG, LDL-cholesterol and TC, and lower HDL cholesterol levels compared with control group [25]. In addition, Mulhim et al. conducted their study to assess metabolic and clinical Profile of Saudi patients with PCOS, they found that PCOS patients had non- significantly difference in TC, HDL cholesterol, TG, LDL cholesterol, or FPG values compared to control group [26].

Regarding association of obesity and PCOS as cardiovascular risk, the study conducted by Stepto et al. demonsterated that the influence of obesity on PCOS clinical and biochemical characteristics was non-significant [27].

Our finding adds to the growing body of evidence implicating that both expression and serum levels of ICAM-1 had an important role in pathogenesis of atherosclerosis as well as PCOS. The current study revealed that ICAM-1 expression and serum levels were significant higher in PCOS patients as compared to control group. Moreover, among PCOS group, T2DM patients had significantly higher expression and serum levels of ICAM-1 in comparison to IGT and NGT. In agreement with our results, Diamanti-Kandrakis et al. proposed that levels of adhesion molecules were higher, including ICAM-1, in PCOS patients compared to healthy women [28].

Similar to our data, Danesh et al. and his colleagues observed that insulin resistance has a central role in chronic inflammation and endothelial dysfunction, which represent an early sign of atherosclerosis [29]. In addition, study conducted by Hak et al. observed higher levels of ICAM-1 in nondiabetic subjects with insulin resistance [30]. Also, regarding the association of T2DM and ICAM-1 expression level, Kado et al. detected higher levels of ICAM-1 expression in hyperglycemia [31]. Similar results found higher adhesion molecules levels in diabetic patients [32, 33],

Our study evaluated the correlations of ICAM-1expression and serum levels, we found that both expression and serum levels were significantly positive correlated with cardiovascular risk and PCOS phenotypes among PCOS patients. Moreover, in order to better elucidate the association of ICAM -1serum levels, linear regression test was done and we observed among clinical and laboratory markers of PCOS, only HOMA-IR was the main predictors of serum ICAM-1 levels in PCOS patients. While, HOMA-IR, WBC count, and ovarian volume were the main predictors of ICAM-1 expression levels among PCOS cases. Similarly, Blankenberg et al. showed positive correlation between cellular adhesion molecules and markers of low-grade inflammation and endothelial dysfunction [34].

A noted feature of PCOS pathogenic process is the role of inflammation. Here presented data revealed higher levels of WBC count and hs-CRP in PCOS patients. Similarly, in earlier published studies, we found that PCOS women had higher levels of inflammatory markers [35, 36] .

One of the main objectives of the current study was to evaluate association between PCOS clinical and laboratory markers with atherosclerosis. We studied CIMT as a marker of pre-clinical atherosclerosis. We found that CIMT was significant high in PCOS patients compared to control group. Moreover, among PCOS group, T2DM patients had significantly higher expression and serum values of CIMT compared to IGT and NGT. In addition, our study revealed significantly positive correlation between CIMT with cardiovascular risk and PCOS phenotypes in PCOS patients. In agreement to our results, Rubio-Guerra et al. CIMT values were positively correlated with circulating adhesion molecules [37]. Similarly, Tardif et al. study suggested that, increased CIMT is independently linked to cardiovascular risks [38]. Notably, also systematic review and meta-analysis confirmed that CIMT could be used diagnostic marker of risk for cardiovascular events [39, 40, 41]. In contrast, Talbott et al. found that after adjusting for age and BMI, PCOS was not a significant predictor of CIMT [42].

In attempt to assess the diagnostic power of ICAM-1 expression and serum levels we evaluated our results using ROC analysis. We observed that the AUC of expression levels of ICAM-1 was higher than serum levels of ICAM-1 in diagnosis of PCOS as well as in discriminating T2DM from NGT patients with PCOS. Interestingly, when we combined diagnostic power of expression and serum levels of ICAM-1 the AUC of ICAM-1 serum levels increased.

## Conclusion

We found that expression and serum levels of ICAM-1were higher in PCOS patients mainly T2DM. Moreover, they were significantly positive correlated with cardiovascular risk and PCOS phenotypes. Interestingly, combination of both ICAM-1 expression and serum levels improved the diagnostic values of serum ICAM-1. thus, the expression and serum levels of ICAM-1could be a useful diagnostic biomarker of PCOS .

## Data Availability

Data available on demand.
